# ICP8-vhs- HSV-2 Vaccine Expressing B7 Costimulation Molecules Optimizes Safety and Efficacy against HSV-2 Infection in Mice

**DOI:** 10.3390/v15071570

**Published:** 2023-07-18

**Authors:** Maria Korom, Hong Wang, Kaelin M. Bernier, Brian J. Geiss, Lynda A. Morrison

**Affiliations:** Department of Molecular Microbiology and Immunology, Saint Louis University School of Medicine, 1100 South Grand Blvd., St. Louis, MO 63104, USA; koromm@gwu.edu (M.K.); hwang360@wustl.edu (H.W.); kaelin.bernier@bannerhealth.com (K.M.B.); brian.geiss@colostate.edu (B.J.G.)

**Keywords:** herpes simplex virus, HSV-2, vaccine, costimulation, genital, antibodies, T cells

## Abstract

Herpes simplex virus 2 (HSV-2) causes most sexually transmitted genital ulcerative disease. No effective prophylactic vaccine is currently available. Replication-defective (ICP8-) HSV stimulates immune responses in animals without producing progeny virus, making it potentially useful as a safe form of a live vaccine against HSV. We previously demonstrated that mice generate a stronger response to ICP8- virus encoding B7-2 costimulation molecules than to the parental replication-defective virus. We have also demonstrated enhanced immunogenicity of an ICP8-, virion host shutoff (vhs)- virus which can no longer destabilize viral and host mRNAs. Here, we constructed a triple mutant, ICP8-vhs-B7-2+ strain, and compared it to both double mutant viruses. Immunization of mice with a single dose of ICP8-B7-2+ or ICP8-vhs-B7-2+ virus decreased challenge virus replication in the vaginal mucosa, genital disease, and mortality more effectively than immunization with the ICP8-vhs- virus. Immunization with ICP8-B7-2+ or ICP8-vhs-B7-2+ virus also effectively suppressed subsequent HSV-2 infection of the nervous system compared to immunization with the ICP8-vhs- virus. ICP8-B7-2+ and ICP8-vhs-B7-2+ strains induced more IFN gamma-producing CD8 T cells and memory CD8 T cells than did ICP8-vhs- virus, potentially explaining the enhanced protective effects. Thus, B7 costimulation molecules expressed from a replication-defective vaccine can enhance vaccine efficacy, even in an immunocompetent host.

## 1. Introduction

Sexually transmitted infections with herpes simplex virus 2 (HSV-2) are the leading cause of genital ulcerative disease. The global burden of HSV-2 infection is staggering, with over half a billion persons affected worldwide [1,2]. HSV-2 infections result in a significant amount of morbidity in the United States; nearly one in five adults have been exposed to HSV-2 [3], and more than 770,000 new infections occur each year [4]. Indeed, the proportion of infected individuals can approach 70% in some demographics [2]. HSV-2 causes ulcerative lesions in anogenital skin and mucosa and is frequently shed in the absence of symptoms. Primary or recurrent infections late in pregnancy pose a significant perinatal risk to babies born to infected mothers. The infected newborn frequently suffers widespread infection with the potential for permanent neurological sequalae and even death. Ulcerative disease associated with HSV-2 also increases the propensity for the acquisition of HIV in exposed individuals. In addition, the psychosocial impacts of recurrent genital infections can be traumatic and isolating. A vaccine to mitigate these infections and prevent transmission is an important, unmet medical need.

Vaccine development against HSV-2 has focused for decades on the development of the potential product with the greatest safety, primarily subunit vaccines composed of viral cell attachment proteins [5]. However, despite showing preclinical promise, a phase III trial of HSV-2 gD in adjuvant modestly reduced oral and genital HSV-1 infections but had no efficacy against HSV-2 [6]. Immune responses to HSV-2 in naturally acquired infections show a great deal of antigenic breadth, encompassing not only surface components of virions important in antibody-mediated neutralization and antibody-dependent cellular cytotoxicity, but also internal structural components and non-structural proteins that are favored in T cell recognition [7,8,9]. In light of decades of experience with glycoprotein vaccine, a new approach that increases the number and type of protein targets and presents them in ways that stimulate the immune system may be essential. Because HSV-2, as with any self-perpetuating species, carries its own mechanisms of defense against immune recognition, the inactivation of critical defenses for maximum immune stimulation may help optimize antiviral immune responses evoked through vaccination.

Live-attenuated viruses as vaccines are a next-generation approach that shows promise. They are more immunogenic than subunit vaccines [10,11,12,13]. Nonetheless, they must be rendered as safe as possible without unduly compromising immunogenicity to prevent risk to vaccinees, particularly persons with potential underlying immune deficits. Some live-attenuated viruses such as ICP0- or gE- HSV-2 [11,14] are effective in protecting animal models, but replication and establishment of latency may be insufficiently attenuated. Viruses lacking a glycoprotein essential for cellular entry are grown in cells that produce a protein that complements the genetic defect. These “single-cycle” viruses undergo one round of replication in the host but cannot initiate a second round. The single-cycle gH- virus is immunogenic and reduces viral recurrences in guinea pigs [15] but proved disappointing in a clinical trial [16]. Strong cellular and humoral immune responses have been achieved using a single-cycle mutant virus ΔgD-2 which protects against large doses of HSV-2 in a variety of models while improving safety over live attenuated viruses [17]. Another form of potentially safer live virus vaccine is deleted in a gene essential for virus replication. It expresses numerous HSV proteins but is replication-defective. One example, *dl*5-29 (UL5-ICP8-) [18], effectively protects against HSV-2 challenge in animal models [12,13,19]. However, this virus grows slowly in culture and requires two large doses to fully protect even mice [12,20]. In addition, *dl*5-29 proved insufficiently immunogenic in phase I trials, especially when administered to seropositive women [21,22]. Newer versions, *dl*5-29-41L or *dl*5-29-41.1 (UL5-ICP8-vhs-) [20,23,24], seek to increase immunogenicity by reducing immune evasion promoted by the virion host shutoff (vhs) protein [25,26]. Although the inactivation of *vhs* in HSV-1 strongly increases immune responses and protection [27], the impact curiously is weaker in the context of the HSV-2 replication-defective vaccine [20].

Independently, the immunogenicity of a live, replication-defective HSV-2 vaccine has been improved by engineering the virus to encode host B7-2 costimulation molecules (ICP8-B7-2+), a critical signal in T cell activation which has been demonstrated to boost T cell responses to vaccination in mice [28]. In addition, B7-2-expressing virus shows strong protective efficacy compared with ICP8- virus [28]. With the goal of optimizing immunogenicity and protective capacity, we sought to determine how these forms of replication-defective viruses compare, attempting to identify which demonstrates the best efficacy while maintaining safety.

## 2. Materials and Methods

### 2.1. Cells and Virus Growth

S2 cells, a Vero cell line stably expressing ICP8 upon infection [29], were used to propagate ICP8-deficient 5BlacZ, 5BΔlacZ and its derivatives. Vero cells were used to generate stocks of HSV-2 strain G-6 [30], a plaque-purified derivative of strain G. For immunizations, the supernatant of infected cell monolayers was collected and subjected to high-speed centrifugation to generate virus free of cell debris as previously described [31]. Virus titers were determined on S2 or Vero cells by standard plaque assay [32]. In experiments requiring HSV-2 strain *dl*5-29 and its derivatives, V529 cells were used because they express ICP8 and UL5 [19,20]. S2 and V529 cells and *dl*5-29 and its derivatives were obtained from David Knipe, Harvard Medical School.

### 2.2. Construction and Isolation of Mutant Viruses

**Figure 1:**
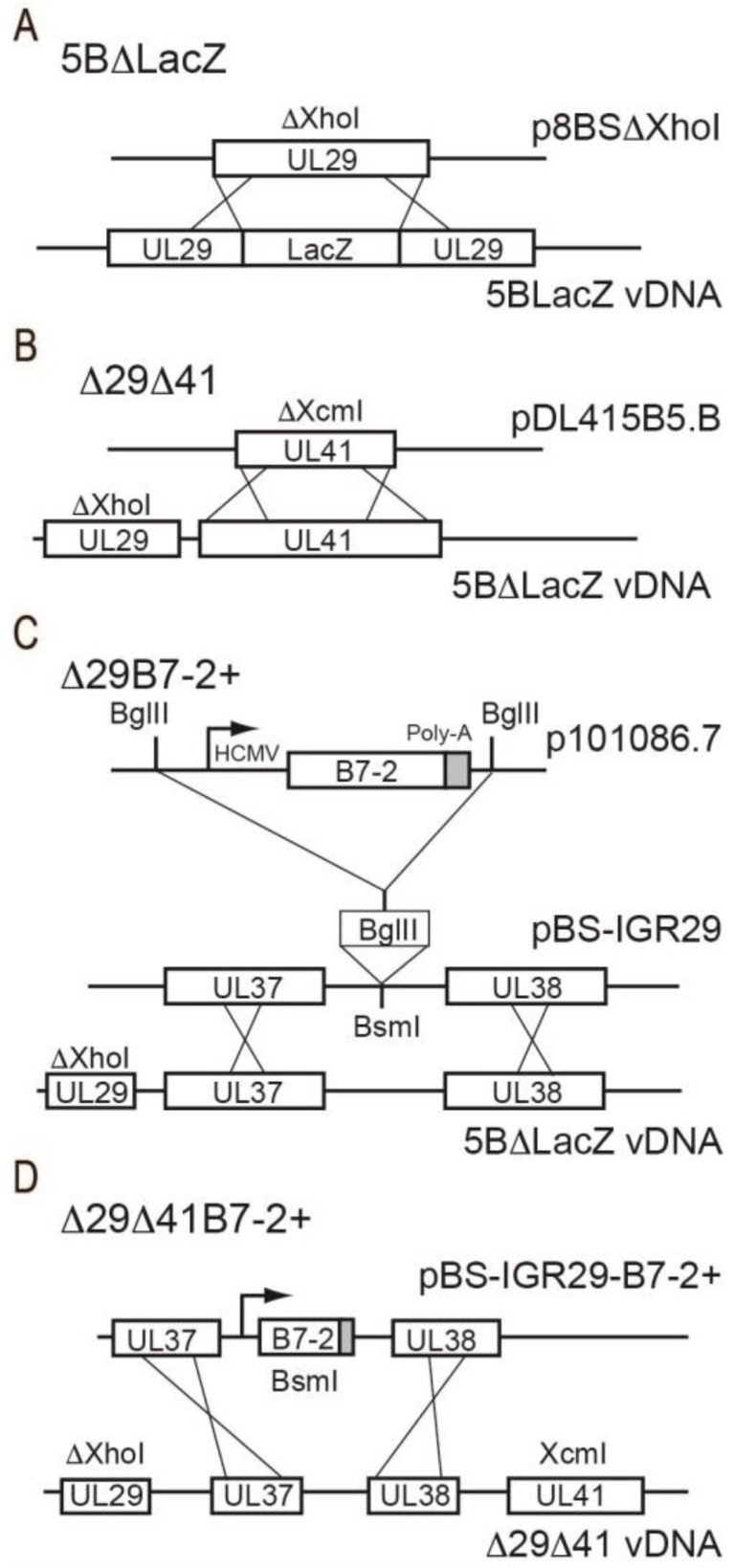
Construction and characterization of recombinant viruses. (**A**) Recombination of XhoI-deleted *UL29* into the DNA of virus 5BlacZ, removing the *lacZ* sequence and generating virus 5BΔLacZ. (**B**) Recombination of XcmI-deleted *UL41* into the DNA of virus 5BΔLacZ, generating virus Δ29Δ41. (**C**) Line 1 shows the insertion cassette containing the HCMV IEp fused to the B7-2 ORF. Line 2 shows insertion of this cassette into the UL38/39 IGR, and line 3 represents recombination of this cassette with 5BΔlacZ viral DNA to generate virus Δ29B7-2+. (**D**) Recombination of the cassette containing the B7-2 ORF (in the UL38/39 IGR) into Δ29Δ41 viral DNA to generate the Δ29Δ41B7-2+ virus.

Certain previously studied, replication-defective HSV-2 vaccine strains also contain a deletion of vhs or encode B7 costimulation molecules [20,23,28]. To facilitate direct comparison of their efficacy we recreated these strains in a homogenous genetic background. The replication-defective mutant, 5BlacZ, does not produce the essential viral gene product ICP8 due to the insertion of the *E. coli lacZ* gene into the UL29 open reading frame [33]. Because lacZ is potentially immunogenic, we mutated 5BlacZ to remove the *lacZ* gene by cotransfection into S2 cells of full-length 5BlacZ DNA along with plasmid p8BSΔXhoI which contains the UL29 open reading frame with an XhoI-XhoI deletion (Figure 1). Plaques under X-gal overlay were screened for white plaques, indicating possible loss of *lacZ* sequences. The identity of candidate recombinant virus 5BΔlacZ, plaque purified to homogeneity, was confirmed by PCR; 5BΔlacZ was used as the basis for all recombinant virus vaccine strains generated in this study. Next, we sought to disrupt *UL41* and/or insert murine CD86 encoding B7-2 costimulation molecules. To engineer a mutation in the *UL41* (*vhs*) ORF of 5BΔlacZ, plasmid pDL41SB5.B containing the *vhs* locus with an XcmI-XcmI deletion was cotransfected with full-length 5BΔlacZ DNA and isolated plaques were screened by PCR for the presence of the deletion in *vhs*. The resulting virus was named Δ29Δ41. To engineer 5BΔlacZ to encode B7-2 costimulation molecules, the UL37/38 intergenic region (IGR) of HSV-2 strain 186 was amplified by PCR and cloned into pBS-KS+ to create pBS-IGR29. A cassette containing the murine B7-2 (CD86) open reading frame driven by the human cytomegalovirus immediate-early enhancer/promoter (IEp) was excised from p101086.7 by BglII digestion and ligated into plasmid pBS-IGR29 which had been modified by insertion of a BglII linker at the BsmI site. The new plasmid, pBS-IGR29-B7-2, was cotransfected with full-length 5BΔlacZ DNA into S2 cells. Cells infected with potential recombinant virus expressing B7-2 costimulation molecules were enriched by panning on Petri dishes coated with anti-B7-2 monoclonal antibody and plaque isolates derived from them were screened by flow cytometry (see below). The identity of a plaque-purified, B7-2-expressing isolate was confirmed by PCR. The resultant virus, Δ29B7-2+, is similar to 5B86 [34], except that 5B86 contains the CD86 cassette inserted into a KpnI-KpnI deletion in the *UL23* thymidine kinase (tk) gene rather than the UL37/38 IGR. The resulting recombinant virus, Δ29Δ41, resembles *dl*5-29-41L [20] except that it contains no *E. coli lacZ* gene and no deletion in the UL5 open reading frame. To engineer a virus containing both the deletion in *vhs* and the CD86 insertion, full-length Δ29Δ41 DNA was cotransfected with plasmid pBS-IGR29-B7-2. Cells infected with the potential recombinant virus were enriched by panning and plaque isolation was performed. Plaque isolates were screened by flow cytometry and a B7-2+ isolate was confirmed by PCR and named Δ29Δ41B7-2+. All recombinant viruses were plaque-purified to homogeneity and the region of their insertion or deletion was verified by sequencing. For ease of labeling figures, the Δ29 designation has been dropped, leaving Δ41, B7-2+, and Δ41B7-2+.

### 2.3. Panning and Flow Cytometry

Petri dishes were coated with anti-mouse B7-2 antibody (BD Biosciences PharMingen, San Diego, CA, USA) (1 µg/mL in 50 mM Tris, pH 9.5) for 1 h at room temperature, and then incubated overnight at 4 °C. Plates were washed extensively with PBS and blocked with 2% newborn calf serum in PBS before use. S2 cells infected with the progeny of a cotransfection (above) were collected by gentle scraping 24 h post-infection and added to Petri dishes at a concentration of 1.2 × 10^6^ cells/plate. After incubation at 37 °C for 1 h, plates were swirled and unbound cells were removed by pipetting. Plates were washed gently with PBS, and then bound cells were scraped into DME + 10% FCS. Collected cells were pelleted and sonicated, and the mixture was diluted for plaque isolation. Plaque isolates were sub-cultured in 24-well plates and collected when CPE reached 100%. A portion of the infected cells from each plaque was pooled with 4 others. The pools were incubated with anti-CD86-PE and analyzed for B7-2 expression by flow cytometry (Figure S1). Each member of a positive pool was then analyzed individually to identify recombinant viruses. Plaque isolates were iteratively purified to homogeneity.

### 2.4. RNA Isolation and Quantitative RT-PCR

Monolayer cultures of 1.5 × 10^6^ to 1.8 × 10^6^ S2 cells were mock infected or infected at a multiplicity of infection (moi) 10 in the presence of 10 µg/mL of actinomycin D (Act D). At 6 h post infection, cytoplasmic RNAs were harvested using an RNeasy Mini kit (Qiagen, Germantown, MD, USA), including the on-column DNase digestion step. RNA Nano Labchips (Agilent, Santa Clara, CA, USA) was used to assess RNA integrity and purity. Five hundred ng of each RNA sample were reverse transcribed using anchored oligo(dT)_18_ primers and a Transcriptor First Strand cDNA Synthesis Kit (Roche, Indianapolis, IN, USA) in 20 µL volume according to the manufacturer’s instructions. Real-time PCR reactions detecting GAPDH mRNA and 18S rRNA were performed on 0.1 µL of cDNA using FastStart SYBR Green Master Mix (Roche) and an ABI 7500 FAST Real-time PCR system (Applied Biosystems, Foster City, CA, USA). Reactions were performed in duplicate in 25 µL volume. For GAPDH, the primers used were 5′-GAACGGGAAGCTTGTCATCAATGG-3′ and 5′-TGTGGTCATGAGTCCTTCCACGAT-3′, which amplify a 343 bp product. For 18S rRNA the primers used were 5′-GTAACCCGTTGAACCCCATT-3′ and 5′-CCATCCAATCGGTAGTAGCG-3′ [35], which amplify a 151 bp product. PCR parameters consisted of 10 min FastStart Taq activation at 95 °C, followed by 40 cycles of 95 °C for 20 s, and 60 °C for 1 min. Specificity was verified by melting curve analysis. GAPDH signal was normalized to the 18S rRNA signal using the 2(−ΔΔCt) method [36,37]. The GAPDH mRNA level in mock-infected S2 cells was set at 100% and the GAPDH mRNA level remaining in virus-infected samples was calculated as a percentage of mock.

### 2.5. Plaque Size Measurement

Infected monolayers incubated for 48 h in the presence of a medium containing human serum were fixed and stained with Giemsa. Plaques were photographed using a Leica DM IRB microscope. The area of 50 randomly selected plaques was determined using Leica Application Suite V4 by tracing around the circumference of each plaque and converting pixels to mm^2^.

### 2.6. Immunizations

Female BALB/c mice were purchased from the National Cancer Institute and were rested for one week before use. BALB.B mice were purchased from the Jackson Laboratory and bred in the Department of Comparative Medicine at Saint Louis University School of Medicine. All mice were housed at Saint Louis University School of Medicine Department of Comparative Medicine under specific-pathogen-free conditions in strict accordance with good animal practice as defined by Institutional and Public Health Service guidelines and with work approved by the Institutional Animal Care and Use Committee.

For immunizations, hind flanks of the mice at 6 weeks of age were injected subcutaneously (s.c.) with a single low (2 × 10^4^ PFU), medium (1 × 10^5^ PFU), or high (5 × 10^5^ PFU) dose of virus suspended in a 40 μL total volume of normal saline. Some mice received an equivalent amount of supernatant concentrated from uninfected cell cultures (control supernatant) as a negative control for immunization. A 30 g needle was used for immunizations to minimize discomfort.

### 2.7. ELISpot Assay

For assessment of T cell responses to immunization, inguinal and paraaortic lymph nodes were harvested from BALB.B mice 6 d after immunization with control supernatant or 1 × 10^5^ PFU of vaccine virus. Single-cell suspensions were prepared, and functional HSV-specific T cells were enumerated by IFN-γ enzyme-linked ImmunoSpot (ELISpot) assay. Lymph node cells (5 × 10^5^ and 2 × 10^5^ cells per well for control supernatant and Δ29Δ41, 4 × 10^5^, and 1.5 × 10^5^ cells per well for Δ29B7-2+ and Δ29Δ41B7-2+) were added to multiscreen-HA plates (Millipore) coated with anti-IFN-γ capture antibody. Cells were cultured in the presence of peptide gB498-505 [38] at 0.2 μM as a CD8 T cell stimulus for 20 h. After the incubation, the membranes were washed, and spots were visualized with an anti-IFN-γ detection antibody, followed by streptavidin-alkaline phosphatase and BCIP (5-bromo-4-chloro-3-indolylphosphate)-nitroblue tetrazolium substrate. An ImmunoSpot plate reader (version 5.0; Cellular Technology, Ltd., Shaker Heights, OH, USA) was used to quantify spots.

IFN-γ ELISpot assay was also employed to assess memory and HSV-specific T cell responses. One month after s.c. immunization, splenocytes were isolated and cultured at the concentrations indicated above with 0.2 μM gB peptide for 20 h on multiscreen-HA plates coated with anti-IFN-γ capture antibody. Spots were developed as described above.

### 2.8. Quantification of Serum Antibodies

To determine the concentration of HSV-specific serum antibodies induced by vaccination, groups of mice were immunized with the vaccine strains or control supernatant or were left unimmunized. Blood was collected from the tail vein of mice 22 d after immunization. The serum remaining after clot retraction was analyzed by enzyme-linked immunosorbent assay (ELISA), as previously described [39]. The secondary antibody used was anti-mouse immunoglobulin G (IgG) biotin (R & D Systems, Minneapolis, MN, USA) which was detected using streptavidin–horseradish peroxide followed by *O*-phenylenediamine dihydrochloride substrate (Sigma-Aldrich, Burlington, MA, USA). Plates were read at 490 nm on a Bio-Rad 680 plate reader. Antibody titers were determined by comparison to standard curves generated with serum containing known concentrations of IgG captured on plates coated with goat anti kappa light chain antibody (Caltag, Burlingame, CA, USA).

To determine the neutralizing activity of antibodies in serum, 2-fold serial dilutions of serum in microtiter plates were mixed with an equal volume containing approximately 50 PFU of HSV-2 G-6 and guinea pig complement (Cedarlane, Burlington, NC, USA; final concentration 1:12) for 2 h at 37 °C. Contents of the wells were then transferred to Vero cell monolayers in 24-well plates and incubated for 1 h at 37 °C. Wells were washed once with PBS and overlaid for standard plaque assay. The neutralizing antibody titer was recorded as the highest serum dilution which reduced plaque number by >50% compared with the control diluent.

### 2.9. In Vivo Challenge

Mice were challenged 4 weeks after immunization. At 7 d and 1 d prior to the challenge, mice were injected s.c. in the neck ruff with 3 mg Depo-Provera (Pfizer, New York, NY, USA) suspended in a 100 μL volume of normal saline. Prior to the challenge, mice were anesthetized by intraperitoneal injection of ketamine/xylazine. Infection occurred by intravaginal (i.vag.) inoculation of 5 × 10^5^ PFU G-6 in a 5 μL volume. To quantify the virus, shed from the genital epithelium, vaginal vaults were swabbed twice with calcium alginate swabs at 9 h and 1 to 5 d post infection. Duplicate swabs for each time point were placed together in 1 mL phosphate-buffered saline and stored frozen until use. The virus was quantified on Vero cell monolayers by standard plaque assay. Body weight, signs of disease, and survival were monitored daily post challenge. Mice were weighed individually, and the mean daily change from initial body weight was calculated for each group. Disease scores were assigned by a masked observer based on the following scale: 0, no apparent signs of disease; 1, slight erythema and edema of the external genitals; 2, prominent erythema and edema of the genitals; and 3, severe erythema and edema with lesions on the genitals. The mean daily disease score was calculated for each group. Hind-limb paralysis was also assessed. Mice were euthanized if they were discovered to have lost more than 20% of their body weight or had become paralyzed. To analyze virus replication in neural tissues, the brains, brainstems, and spinal cords were dissected from a cohort of mice 5 d after the challenge. Tissues were stored frozen until use. The tissues were subsequently thawed and disrupted using a mini-bead beater (BioSpec, Bartlesville, OK, USA), and then diluted for standard plaque assay.

### 2.10. Statistics

The significance of the difference in antibody concentrations and virus titers on individual days was determined by ANOVA with Bonferroni correction, as was the difference in the number of IFN-γ-producing T cells. Proportions of mice with hind-limb paralysis were compared using the Fisher exact method. The Mann–Whitney U nonparametric test was used to assess the significance of the difference in disease scores on individual days post challenge.

## 3. Results

### 3.1. Construction, Isolation, and Characterization of Recombinant Viruses

Previous replication-defective HSV-2 vaccine strains varied by several parameters: 5B86 [34] contains the *E. coli* lacZ gene in the ICP8 locus and is tk- due to the insertion of CD86 (encoding B7-2). *dl*5-29-41L [20] contains *lacZ* in the *vhs* locus and a deletion in UL5. To avoid the potentially misleading effects of these differences and allow for legitimate direct comparison, we built a new set of replication-defective vaccine strains. First, we replaced the *lacZ*-disrupted ICP8 locus in 5BlacZ (Figure 1A) with an ICP8 ORF containing a deletion known to interfere with the ICP8 function (Figure 1B). This virus, 5BΔlacZ, contains no bacterial genes and formed the basis for all our ICP8-, replication-defective vaccine strains. To create a *dl*5-29-41L counterpart, we replaced the *vhs* open reading frame in 5BΔlacZ with a version that contains a deletion (Figure 1C) known to inactivate vhs [40]. This virus was named Δ29Δ41. To create a 5B86 counterpart, we ligated a CD86 (B7-2) expression cassette [34] into the UL37/UL38 IGR (Figure 1D), which is neutral with respect to HSV-2 replication and virulence (Korom and Morrison, unpublished result). This virus was named Δ29B7-2+. Finally, to combine the alterations to 5BΔlacZ, we replaced the *vhs* ORF in Δ29B7-2+ with *vhs* containing the deletion, thus creating virus Δ29Δ41B7-2+ (Figure 1D). All viruses were plaque-purified and their modifications were verified by sequencing the relevant region(s).

**Figure 2:**
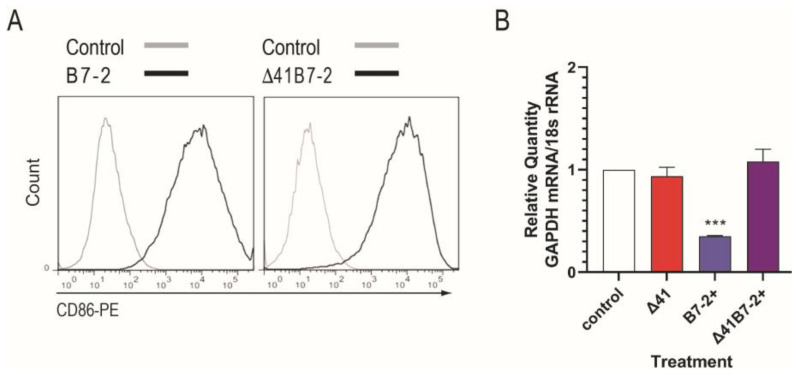
B7-2 expression and vhs assay. (**A**) S2 cells were mock-infected or infected with Δ29B7-2+ or Δ29Δ41B7-2+ at moi of 5 for 18 h, then collected, stained with anti-CD86-PE, and analyzed by flow cytometry. (**B**) S2 cells were mock-infected or infected with the indicated viruses at moi 10. Six hours post infection, mRNA was isolated, reverse transcribed, and GAPDH expression was analyzed by real-time PCR. The GAPDH signal was normalized to the 18S rRNA signal, the GAPDH mRNA level in mock-infected Vero cells was set at 100%, and the GAPDH mRNA level in virus-infected samples was calculated as a percentage of mock. Results are from one of 2 independent experiments performed. ***, *p* = 0.0021.

Vero cells infected with Δ29B7-2+ and Δ29Δ41B7-2+ expressed B7-2 costimulation molecules on their surface as detected by flow cytometry (Figure 2A). To analyze vhs activity in the mutant viruses we conducted quantitative reverse transcriptase real-time PCR using primers for GAPDH and cDNA template prepared from mRNA of cells 6 h post-infection. Cells infected with Δ29Δ41and Δ29Δ41B7-2+ contained more GAPDH mRNA than cells infected with Δ29B7-2+ which has an intact *vhs* gene (Figure 2B), verifying that the XcmI-XcmI deletion reduced vhs RNase activity (Figure 2B).

**Figure 3:**
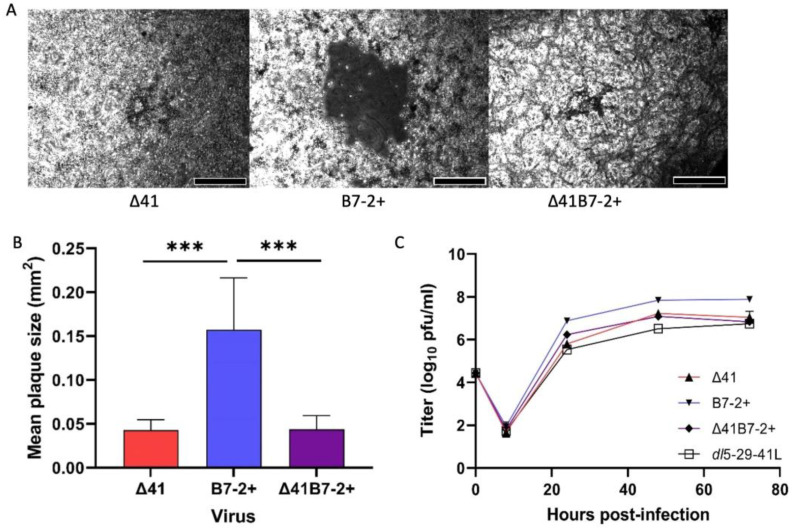
Plaque size comparison. S2 cells were infected with the indicated viruses and incubated for 48 h before fixation and staining. Plaques were photographed at 50× magnification. (**A**) Representative plaques are shown. Scale bar = 200 µm. (**B**) Plaque sizes were calculated using ImageJ software. Data represent the mean ± SD (*n* = 50 for each virus). ***, *p* < 0.001; Δ41 v. Δ41B7-2+, not significant. (**C**) V529 monolayers were infected at moi 0.3 in duplicate, then scraped and collected at incremental times post infection. Virus titers were determined on fresh V529 cell monolayers.

Interestingly, the *vhs* deletion resulted in smaller plaque size for both Δ29Δ41 and Δ29Δ41B7-2+ compared with Δ29B7-2+ (Figure 3A,B) and resulted in lower titers on a complementing cell line (Figure 3C).

### 3.2. Effect of Immunization with Recombinant Viruses on Protection from HSV-2 Challenge

**Figure 4:**
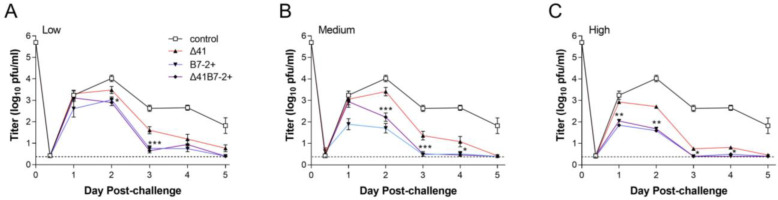
Titers of challenge virus shed from the genital mucosa. Groups of 10 BALB/c mice in each of two independent experiments were immunized once s.c. with a single (**A**) low (2 × 10^4^ PFU), (**B**) medium (1 × 10^5^ PFU), or (**C**) high (5 × 10^5^ PFU) dose of supernatant-derived virus or an amount of control supernatant (control) equivalent to the high dose of virus. All mice were challenged 1 month after immunization by i.vag. infection with HSV-2 strain G-6. Titers of virus collected by vaginal swab of 6 mice per group (*n* = 12 total) at the indicated times post infection were determined by standard plaque assay. Data represent the geometric mean ± SEM for the compiled samples. *, *p* = 0.049–0.01; **, *p* = 0.009–0.001; ***, *p* = 0.0009–<0.0001 for Δ41 compared with Δ41B7-2+.

We had previously demonstrated effective protection of mice from HSV-2 challenge after a single low-dose immunization with 5B86, a virus similar to Δ29B7-2+ but expressing β-galactosidase. *dl*5-29-41L is functionally equivalent to Δ29Δ41, being replication-defective and having *vhs* disrupted. We sought to compare the efficacy of vhs- to B7-expressing virus and to determine whether the provision of B7-2 in the Δ29Δ41 background would increase protective capacity. Thus, we immunized groups of BALB/c mice once with a low, medium, or high dose of Δ29Δ41, Δ29B7-2+, or Δ29Δ41B7-2+ virus, or control supernatant of uninfected cells. Mice were then challenged i.vag. one month later with a heterologous wild-type strain of HSV-2. Over the first 4 d post challenge, Δ29B7-2+ and Δ29Δ41B7-2+ immunizations reduced challenge virus replication in the genital mucosa to a greater extent than Δ29Δ41 (Figure 4).

The differences in replication were significant on days 2 and 3 in mice immunized with the low dose (Figure 4A) and on days 1 through 4 in the high-dose immunization group (Figure 4C). In mice immunized with the medium dose (Figure 4B), only Δ29B7-2+ reduced challenge virus replication on the first day after the challenge, but both Δ29B7-2+ and Δ29Δ41B7-2+ viruses reduced challenge virus replication in the mucosa significantly better than Δ29Δ41 on days 2 through 4. By 5 d post challenge, replication of the challenge virus was still robust in the genital mucosa of control mice, but all vaccine strains had limited replication to barely detectable levels (Figure 4).

**Figure 5:**
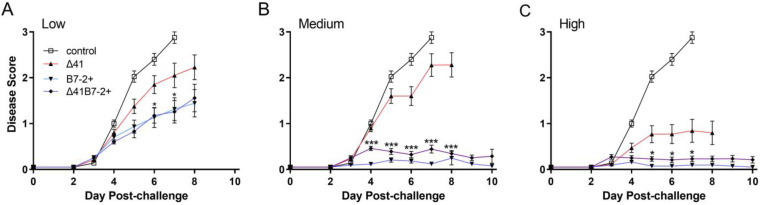
Genital inflammation and disease after i.vag. challenge of immunized mice. Mice as described in Figure 4 were immunized with a (**A**) low, (**B**) medium, or (**C**) high dose of virus or an amount of control supernatant corresponding to the high dose of virus. After the challenge, mice were observed daily for signs of inflammation and lesions on their external genitalia. Data represent the arithmetic mean ± SEM for all samples compiled from two independent experiments (*n* = 20). *, *p* = 0.0389–0.0178; ***, *p* < 0.0001 for Δ41 compared with Δ41B7-2+.

In control mice, HSV-2 induced severe inflammation of the genital mucosa and lesions, whereas prior immunization with any of the vaccine strains limited genital inflammation. Δ29B7-2+ and Δ29Δ41B7-2+ viruses protected mice better than Δ29Δ41 at all three immunizing doses tested (Figure 5).

**Figure 6:**
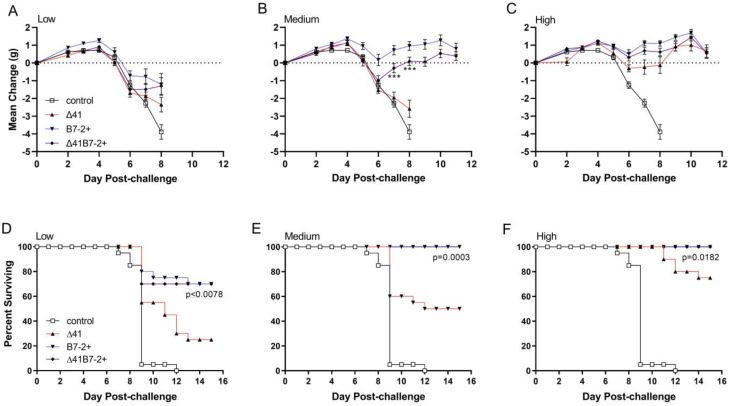
Body weight and survival after i.vag. challenge of immunized mice. Mice as described in Figure 4 were immunized with the low, medium, or high dose of virus or an amount of control supernatant corresponding to the high dose of virus. Body weight (**A**–**C**) and survival (**D**–**F**) were monitored over time after the challenge. Weight data represent the arithmetic mean ± SEM for all mice compiled from two independent experiments (*n* = 20). ***, *p* = 0.0002–<0.0001 for Δ41 compared with Δ41B7-2+.

The difference between viruses expressing B7-2 and the Δ29Δ41 group was most significant after medium dose immunization, with protection from genital inflammation significantly enhanced beginning 4 d post challenge (Figure 5B); however, significantly enhanced protection was also observed at the low and high vaccine doses (Figure 5A,C). Using maintenance of body weight as an indicator of general health, we observed that low-dose immunization with any of the vaccine strains did not protect mice from weight loss after the challenge (Figure 6A).

In mice receiving medium dose vaccine, differences in body weight over time post-challenge reflected differences in genital inflammation and disease in that those mice immunized with Δ29B7-2+ or Δ29Δ41B7-2+ showed only a transient decrease in body weight, whereas those immunized with Δ29Δ41 lost weight at a rate similar to control mice (Figure 6B). All vaccine strains, when given at the high dose, significantly protected mice from weight loss after the challenge compared with the control group (Figure 6C). Consistent with the weight loss profile, most mice receiving the low dose of Δ29B7-2+ or Δ29Δ41B7-2+ vaccine survived challenge virus infection, but most mice immunized with low-dose Δ29Δ41 succumbed (Figure 6D). Deaths occurred in Δ29Δ41-immunized mice at the medium and high doses as well, but all mice immunized with Δ29B7-2+ or Δ29Δ41B7-2+ viruses survived the challenge (Figure 6E,F). Mortality resulting from challenge virus infection was associated with neurological debilitation (Table 1). The medium and high doses of Δ29B7-2+ and Δ29Δ41B7-2+ vaccines protected mice completely from hind-limb paralysis, but a portion of mice immunized with Δ29Δ41 became paralyzed from challenge virus infection.

**Figure 7:**
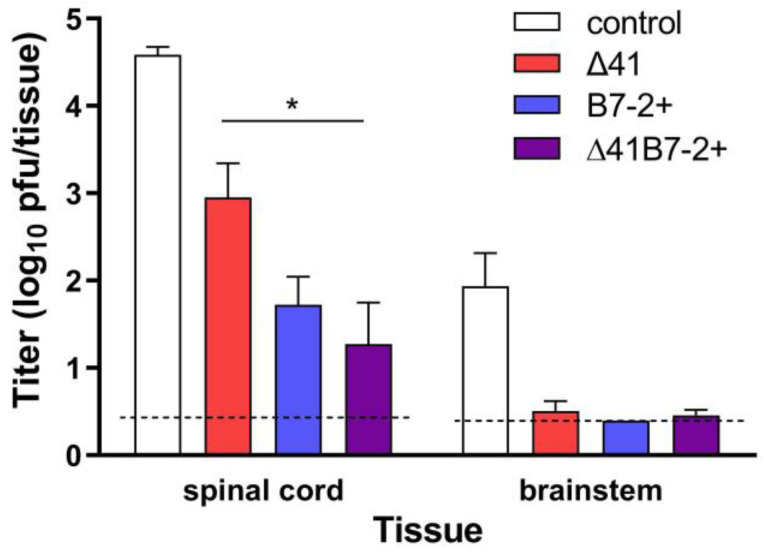
Levels of challenge virus in the nervous system. Mice were immunized with the medium dose of the indicated virus or control supernatant and challenged i.vag. one month later with HSV-2 G-6. After 5 d the mice were euthanized, the indicated regions of the CNS were dissected and homogenized, and the virus titer in them was determined by standard plaque assay. Data represent the geometric mean ± SEM for all samples per group, compiled from 3 independent experiments (*n* = 10 to 12). *, *p* < 0.05.

Hind-limb paralysis and death strongly suggested entry of challenge virus into the nervous system from the genital mucosa. To address this possibility, additional groups of mice were immunized with the medium dose of vaccine viruses, which was the dose demonstrating the greatest differences in genital and neurological signs of disease. Five days after i.vag. challenge, the titer of wild-type HSV-2 in regions of the nervous system was determined (Figure 7).

Compared with mice receiving control supernatant, prior immunization with Δ29Δ41 resulted in less challenge virus in the spinal cords (*p* = 0.014). Immunization with viruses expressing B7-2 further reduced the amount of challenge virus detectable in the spinal cord. Almost no challenge virus was detected in the brainstem of mice immunized with any of the vaccine strains at 5 d post challenge, though the challenge virus had already reached the brainstem in control mice (*p* = 0.0009–0.0002). Thus, all the vaccine strains provided significant protection against acute infection of the nervous system, with the best protection afforded by viruses expressing B7-2.

### 3.3. Immune Correlates of Protection

**Figure 8:**
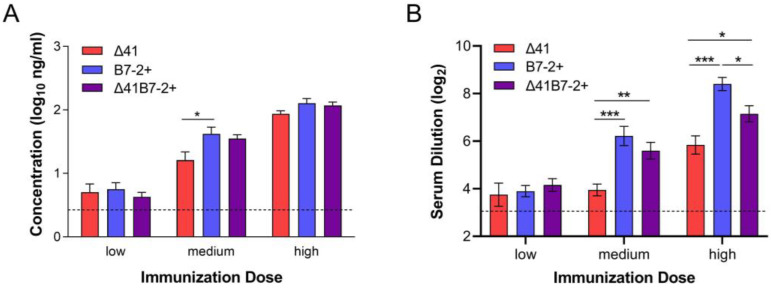
HSV-specific antibody in the sera of immunized mice. Serum samples were collected 22 d after immunization of mice receiving low, medium, or high doses of virus. (**A**) Concentration of HSV-specific IgG was determined by ELISA. (**B**) Neutralizing antibody titer was determined by plaque inhibition assay. Data represent the geometric mean ± SEM of 20 samples compiled from two independent experiments (*n* = 20 total). *, *p* = 0.027–0.0157; **, *p* = 0.0023; ***, *p* < 0.0001.

To define immune responses that correlated with protection mediated by the Δ29B7-2+ and Δ29Δ41B7-2+ viruses, we analyzed HSV-2-specific antibodies and T cell responses stimulated by vaccination. HSV-2-specific serum IgG was evoked by vaccination in a dose-dependent manner (Figure 8A).

**Figure 9:**
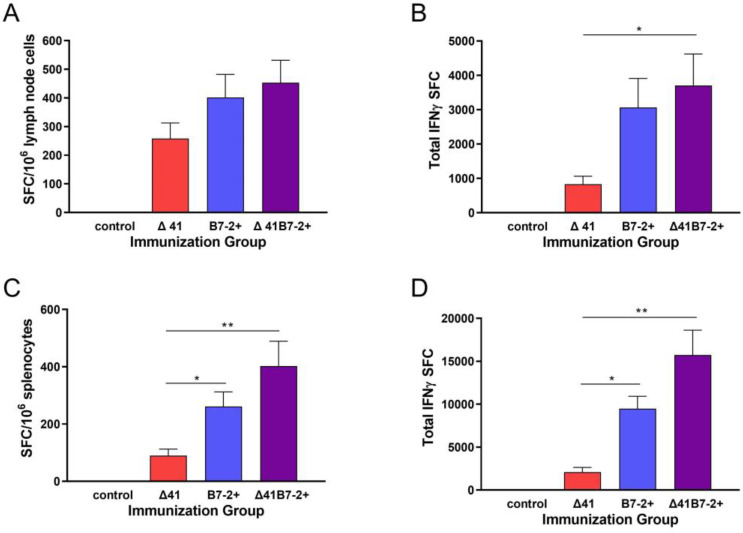
IFN-γ-producing T cells induced by immunization. Groups of BALB.B mice were immunized with 1 × 10^5^ PFU of the indicated replication-defective virus or control supernatant. Cells from the draining lymph nodes were isolated 6 d after immunization, stimulated in vitro with 0.2 mM of peptide representing the CD8 epitope gB498–505, and analyzed in an IFN-γ ELISpot assay. Data represent the arithmetic mean ± SEM of (**A**) spot-forming cells (SFC) per million lymph node cells or (**B**) the absolute number of IFN-γ-producing SFC in the draining lymph nodes per mouse. Data were compiled from 3 independent experiments (*n* = 9 for the control group and *n* = 11 for each vaccine group). Alternatively, splenocytes were isolated 1 mo after immunization and stimulated as above, then analyzed in the IFN-γ ELISpot assay to determine (**C**) SFC per million splenocytes or (**D**) the absolute number of SFC per mouse. Data were compiled from 3 independent experiments (*n* = 7 for the control group and *n* = 8 to 9 for each vaccine group). *, *p* = 0.0475–0.0212; **, *p* = 0.0016; ***, *p* < 0.0001.

All vaccine strains induced equivalent virus-specific IgG responses in serum at the low dose, but the medium dose of B7-expressing viruses stimulated a slightly more robust response (Figure 8A). The same sera were used to test the capacity to neutralize virus infectivity. Interestingly, neutralizing antibodies developed to the greatest extent after immunization with the Δ29B7-2+ virus (Figure 8B). The capacity of the vaccine viruses to stimulate HSV-specific T cell responses was analyzed 6 d after immunization by IFN-γ ELISpot. BALB.B mice were immunized to take advantage of an immunodominant, CD8 T cell epitope recognized by H-2^b^-haplotype mice [38]. Immunization with the Δ29B7-2+ and particularly the Δ29Δ41B7-2+ virus elicited more IFN-γ-producing, CD8 T cells than Δ29Δ41 in the draining lymph nodes 6 d later (Figure 9A), with a significant difference observed when the total number of IFN-γ-producing, CD8 T cells was considered (Figure 9B).

Memory CD8 T cells, however, were markedly more prevalent in the spleens one month after immunization of mice with Δ29B7-2+ or Δ29Δ41B7-2+ than with Δ29Δ41, whether measured as epitope-specific cells per 10^6^ cells (Figure 9C), or the total number of epitope-specific cells per spleen (Figure 9D). Thus, more HSV-specific, memory CD8 T cells were available at the time of the challenge in mice immunized with Δ29B7-2+ and Δ29Δ41B7-2+.

## 4. Discussion

An effective vaccine against HSV-2 must be safe while simultaneously stimulating strong and effective immune responses. Various alterations of replication-defective viral genomes such as disruption of vhs activity or inclusion of B7 costimulation molecules have resulted in more immunogenic and effective vaccine strains than their predecessor strains [20,27,28,34,41]. Direct comparison of the efficacy of these manipulations to the basic replication-defective HSV-2 vaccine paradigm, however, demands that extraneous differences be minimized. Therefore, we constructed all vaccine strains in the same replication-defective (ICP8-deleted) background to evaluate the effectiveness of the virus with vhs deleted to those expressing B7-2. Furthermore, these viruses lacked any bacterial gene (*lacZ*) that could itself be immunogenic and potentially influence anti-viral immune induction. The central finding of our comparison is that replication-defective viruses expressing B7 costimulation molecules are superior in protective efficacy to replication-defective, vhs- viruses.

As was previously observed [24], viruses bearing a deletion in HSV-2 *UL41* had a smaller plaque phenotype on complementing cells than viruses capable of expressing vhs. This correlated with a reduction of maximal titer that could be achieved on complementing cells, an observation that bears on the ability to generate a sufficient quantity of a live virus vaccine for manufacture. Nonetheless, the deletion of *UL41* has benefits for vaccine design. Compromise of vhs activity enhances recognition of infected fibroblasts [42] and relieves the block to activation in infected dendritic cells, potentially a critical feature of a whole virus vaccine [26]. Interestingly, loss of vhs affects replication-defective HSV-2 more severely than it does HSV-1 strains, which may reflect the stronger, faster activity of HSV-2 vhs previously described [43,44,45,46]. Indeed, Reszka et al. [24] showed that the substitution of HSV-1 vhs into *dl*5-29 resulted in higher virus yield than *dl*5-29Δ41 but did not affect its immunogenicity. The vhs protein is also a target of HSV-immune T cells [7]. Potentially the Δ29Δ41B7-2+ vaccine strain could be further optimized by creating a smaller deletion in *UL41* which preserves most of the protein as an immunologic target while still compromising vhs activity, or by doing so in a substituted HSV-1 *UL41* gene.

Comparison of these viruses as vaccines in mice yielded several interesting observations. First, in a previous study, Hoshino et al. had shown that replication-defective, vhs- HSV-2 vaccine only transiently extended the survival time of mice compared to mice immunized with replication-defective virus alone [23]. Here, we confirmed that deletion of vhs did not confer any consistent, additional protective advantage for Δ29Δ41B7-2+ compared with Δ29B7-2+, though it did increase safety by creating a major insult at a second locus. Second, expression of B7-2, whether in the context of ICP8- virus or ICP8-vhs- virus, increased protective efficacy as measured by HSV-2 shed from the genital mucosa of mice, signs of genital inflammation and disease, infection of the nervous system, and maintenance of body weight and survival. Third, the medium dose of vaccine most clearly distinguished the vaccine strains. While all showed a pronounced capacity to protect at the high immunization dose compared with control vaccination, at the medium dose the Δ29Δ41 virus more closely resembled control immunization, particularly in terms of disease progression. Because of this ability to distinguish between the vaccine strains, we chose the medium dose to investigate the vaccine’s capacity to protect against acute infection of the nervous system. Once again, the B7-expressing viruses could be distinguished from Δ41Δ29 in heightened protection against infection of the spinal cord by 5 d post challenge. Lastly, a single dose of B7-2-expressing vaccine provided significant protection against some aspects of challenge virus infection in mice compared with control supernatant or vhs- virus, even when administered at the lowest dose. Whether these vaccine strains can protect mice from the establishment of latency by challenge virus is a worthy future direction. The establishment of latency by the vaccine strains themselves was not investigated here because DNA of analogous replication-defective strain *dl*5-29 was not detected in sensory ganglia after intramuscular or intradermal immunization, and only low copy numbers were observed after intranasal immunization of mice [19]. Nonetheless, this point must be investigated with B7-expressing strains in subsequent trials to firmly establish their safety profile.

Consistent with the role of B7 costimulation molecules in potentiating differentiation of naïve T cells in response to antigen, we observed a substantial increase in the CD8 T cell response to viruses that express B7-2. Interestingly, this enhancement was most prominent in the memory T cell response rather than in the acute phase. The difference between acute and memory phases could be a result of the particular day after immunization that we looked at and/or to a cytokine milieu that drives initial T cell expansion in response to Δ29Δ41 but does not support efficient conversion to or expansion of memory cells. A similar enhancement in nascent CD8 T cell responses to an ICP8-vhs-B7+ HSV-1 strain compared with the ICP8-vhs- virus was previously observed [41]. Further investigation into the mechanism by which virus-expressed B7 molecules enhance responses to the vaccine is warranted, particularly the cell type(s) in which B7 is expressed, and those that can act as antigen-presenting cells to potentiate the immune response.

## Figures and Tables

**Table 1:** Percentage of mice with hind-limb paralysis.

	Immunization Group			
Vaccine Dose	Control	Δ41	B7-2+	Δ41B7-2+
Low	nd ^a^	50	30	35
Medium	nd	50	0 ^b^	0
High	75	20	0	0

^a^ nd, not done. ^b^ *p* = 0.0004 compared with Δ29Δ41.

## Data Availability

Underlying original data are available upon request.

## Supplementary Materials

The following supporting information can be downloaded at: https://www.mdpi.com/article/10.3390/v15071570/s1, Figure S1: iterative isolation of B7-expressing virus by panning of infected cells and flow cytometry.
