# Supplementary material for: ICP8-vhs- HSV-2 Vaccine Expressing B7 Costimulation Molecules Optimizes Safety and Efficacy against HSV-2 Infection in Mice
Source: Viruses. 2023 Jul 18;15(7):1570. doi: 10.3390/v15071570 (PMC10384616; doi:10.3390/v15071570)
Supplement: Supplementary file 1 [file viruses-15-01570-s001.zip › viruses-2460373-supplementary.pdf]

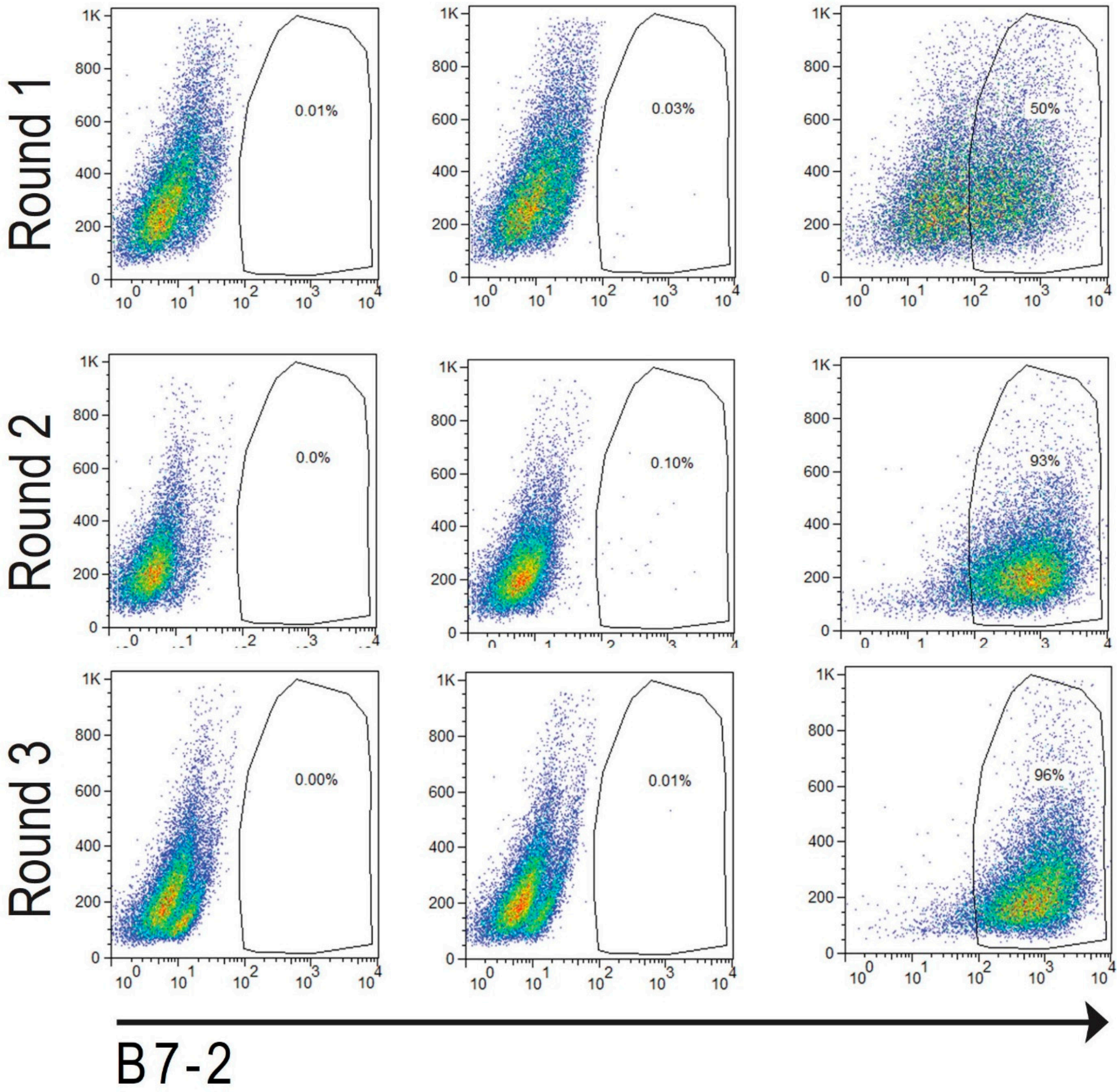

Figure S1. Iterative isolation of B7-expressing virus by panning of infected cells and flow cytometry. Plaques of potential recombinant virus were isolated from cells that adhered to a plate coated with anti-B7-2. A portion of infected cells from 5 plaque picks was pooled, stained with fluorescent antibody to B7-2 and analyzed by flow cytometry. Round 1 shows the results of three such pools. Plaques from the pool containing B7-expressing cells were grown and analyzed individually. Round 2 shows the results of three independent plaques that made up the Round 1 positive pool. The B7-expressing isolate identified in Round 2 underwent another round of plaque purification and was reanalyzed. The results are shown in Round 3, along with two other negative plaque isolates for comparison.
